# aaHash: recursive amino acid sequence hashing

**DOI:** 10.1093/bioadv/vbad162

**Published:** 2023-11-11

**Authors:** Johnathan Wong, Parham Kazemi, Lauren Coombe, René L Warren, Inanç Birol

**Affiliations:** Canada’s Michael Smith Genome Sciences Centre, BC Cancer, Vancouver, BC V5Z 4S6, Canada; Canada’s Michael Smith Genome Sciences Centre, BC Cancer, Vancouver, BC V5Z 4S6, Canada; Canada’s Michael Smith Genome Sciences Centre, BC Cancer, Vancouver, BC V5Z 4S6, Canada; Canada’s Michael Smith Genome Sciences Centre, BC Cancer, Vancouver, BC V5Z 4S6, Canada; Canada’s Michael Smith Genome Sciences Centre, BC Cancer, Vancouver, BC V5Z 4S6, Canada

## Abstract

**Motivation:**

*K*-mer hashing is a common operation in many foundational bioinformatics problems. However, generic string hashing algorithms are not optimized for this application. Strings in bioinformatics use specific alphabets, a trait leveraged for nucleic acid sequences in earlier work. We note that amino acid sequences, with complexities and context that cannot be captured by generic hashing algorithms, can also benefit from a domain-specific hashing algorithm. Such a hashing algorithm can accelerate and improve the sensitivity of bioinformatics applications developed for protein sequences.

**Results:**

Here, we present aaHash, a recursive hashing algorithm tailored for amino acid sequences. This algorithm utilizes multiple hash levels to represent biochemical similarities between amino acids. aaHash performs ∼10× faster than generic string hashing algorithms in hashing adjacent *k*-mers.

**Availability and implementation:**

aaHash is available online at https://github.com/bcgsc/btllib and is free for academic use.

## 1 Introduction

Analysing proteins provides opportunities to elucidate more direct insights into the biochemical pathways and functional activities of cells, tissues, and organisms compared to analysing nucleic acids alone. Conservation at the protein level can reveal important functional and evolutionary insights that may not be immediately apparent when studying sequences at the nucleotide level because of codon degeneracy (Miyata *et al.* 1980). For example, in an assessment of antimicrobial resistance, counting amino acid *k*-mers as opposed to nucleotide *k*-mers was shown to enable higher accuracy and enhanced interpretability of machine learning algorithms (ValizadehAslani *et al.* 2020).

While there are nucleotide-specific hashing algorithms designed for hashing *k*-mers (Mohamadi *et al.* 2016, Kazemi *et al.* 2022, Pibiri *et al.* 2023), there is no stand-alone and optimized implementation that leverages the characteristics of protein *k-*mers to the best of our knowledge. These nucleotide-specific algorithms first break the sequences into *k*-mers and, typically, map them to 64-bit integers, the largest native type supported by computers. Mapping is achieved by using a hashing algorithm such as ntHash (Mohamadi *et al.* 2016, Kazemi *et al.* 2022), or by encoding nucleic acid characters, Σ = {A, C, G, T|U} using two bits (00, 01, 10, 11) (Simpson *et al.* 2009). Compared to using a hashing algorithm, 2-bit encoding has the advantage of being reversible, but is limited to *k-*mers of length 32 bp or shorter using a single 64-bit register. This length limitation is acceptable for certain genomic applications, such as mapping (Li 2018), polishing (Li *et al.* 2022), or scaffolding (Coombe *et al.* 2021, 2023) but not for other applications, such as de Bruijn graph genome assembly for complex organisms (Jackman *et al.* 2017).

Similarly, amino acids can utilize a 4- or 5-bit encoding, a variant of 2-bit encoding, but this can only capture *k*-mers that are up to 16 amino acid residues in length or shorter using 64 bits, thereby virtually leaving general hashing algorithms as the only viable alternative for hashing longer peptide *k*-mers. Moreover, unlike nucleic acid sequences, where every base substitution is considered equally likely with the use of an identical penalty for any nucleotide mismatch in sequence alignment algorithms (Smith and Waterman 1981), the interrelationships between amino acids are more complex, necessitating an understanding of molecular structure and biochemical properties, such as hydrophobicity, polarity, and pKa, a measure of the acidity of a molecule (also known as the dissociation constant). The BLOSUM62 (Henikoff and Henikoff 1992) matrix incorporates the relationships between amino acids and is used by algorithms such as BLASTp (Altschul *et al.* 1990) to score protein–protein alignments. This matrix quantifies the likelihood of a substitution event between two amino acids based on observed mutations in protein sequences that are no more than 62% identical. Hash seeds generated using the BLOSUM62 matrix have been shown to improve sensitivity in homology search (Li *et al.* 2009). With high-throughput protein or peptide sequencing platforms on the horizon, researchers will need fast and efficient algorithms tailored for amino acid sequences to rapidly exploit the influx of data and expedite their analyses (Alfaro *et al.* 2021).

In recent years, a number of tools that use amino acid *k-*mers have been developed. KAAmer (Déraspe *et al.* 2022) is a database that utilizes key-value pairs to link amino acid 7-mers, which have been hashed to their corresponding 32-bit hash values by combining the hash values of each constituent amino acid with bitwise XOR and shift operations, to the locations of their corresponding contents, i.e. proteins that contain the *k*-mers. Linclust (Steinegger and Söding 2018), a linear-runtime algorithm designed to efficiently cluster vast metagenomic datasets, clusters sequences by first finding shared amino acid *k-*mers through hashing. The formulation of Linclust’s hash function is similar to that of KAAmer, but it also incorporates a cyclical hashing component, eliminating the need to rehash the entire *k-*mer when hashing consecutive *k*-mers. SECOM (Fan *et al.* 2012) is a domain prediction method that uses hash seeds (Li *et al.* 2009) to index proteins and employs community detection to identify protein domains. To generate the hash seeds, SECOM utilizes the Rabin–Karp (Karp and Rabin 1987) rolling hash algorithm. It first assigns each amino acid to an integer representation based on the classification setting, then multiplies it by a base raised to the power of its position, sums the results, and finally applies a modulus operation to the final hash value. Miniprot (Li 2023), a protein to genome aligner, uses the *k-*mers from a query protein to identify alignment anchors by querying a 6-frame translated genome index using a 4-bit encoding scheme where multiple amino acids are mapped to the same hash value. An optimized amino acid hashing algorithm that does not suffer from the restrictions of 4- or 5-bit encoding could boost the performance of these tools by reducing the processing time and capturing more specific amino acid *k*-mers. Here we present aaHash, a hashing algorithm designed for amino acid sequences that uses multi-level seed tables to represent the biochemical similarities between amino acids.

## 2 Methods

aaHash builds on ntHash (Mohamadi *et al.* 2016, Kazemi *et al.* 2022), a rolling hash algorithm for DNA/RNA sequences, and adapts it for amino acid sequences. Similar to ntHash, aaHash utilizes a seed table to rapidly hash adjacent *k*-mers. This seed table contains 20 64-bit integers indexed using the amino acids alphabet, Σ = {A, C, D, E, F, G, H, I, K, L, M, N, P, Q, R, S, T, V, W, Y}. The 20 64-bit integers were chosen to ensure a balanced bit distribution, with each having a nearly equal number of 1s and 0s in their binary representation. aaHash also employs pre-built amino acid dimer and trimer seed tables to expedite the calculation of the initial hash value (input to recursive hash value calculations) (Supplementary Fig. S1).

To leverage the amino acid relationships captured in the BLOSUM62 matrix, we first transformed the matrix such that the positive scores (Supplementary Fig. S2), indicating molecular similarity, are concentrated along the diagonal, thereby grouping similar amino acids together (Li *et al.* 2009). We then determined different zones of degeneracy where all the amino acids within the zone should evaluate to the same hash. Using these zones, we created three levels of hashes for aaHash by having seed tables for each level of hash. The first level is a hash function where each amino acid character corresponds to a 64-bit hash value, the default behaviour in generic string hashing. For level 2, we implemented a degenerate hashing scheme where amino acids are grouped when they have positive scores with each other in the BLOSUM62 matrix. Similarly, for level 3, we expanded the definition of degeneracy to include amino acids with nonnegative scores. For hash levels 2 and 3, amino acids in the same degeneracy group will all evaluate to the same hash value. aaHash also supports using these different levels of hashes together to create a multi-level pattern, mimicking the functionality of spaced seeds (Ma *et al.* 2002).

The base formula for aaHash is defined in Equation (1). In this equation, *H* is the resulting aaHash hash value, *s* represents the sequence (*k-*mer) being hashed, *k* refers to the size of the *k*-mer, *h* is the lookup table that maps each amino acid to a 64-bit integer, and finally *l* defines the level of hashing.


(1) $$\begin{array}{c} H\left(s_{0}\right)=\mathrm{sro}l^{k-1}(h_{l}\left(s_{0}\left[0\right]\right))\;⊕\;\mathrm{sro}l^{k-2}(h_{l}\left(s_{0}\left[1\right]\right))\;⊕\;\ldots \;⊕\\ \;h_{l}\left(s_{0}\left[k-1\right]\right) \end{array}$$

The srol operation, which denotes a cyclical bit-rotate-and-swap, is shown in Equation (2). This operation incorporates a bit swap mechanism between the 31st and 33rd bits using the swap_bits function and a bitwise left-rotate operation with rol to increase the period of rotations from 64 to 1023.


(2) $$\mathrm{srol}\left(x\right)=\mathrm{swap}\_\mathrm{bits}\left(\mathrm{rol}\left(x\right)\right)$$

The recursive formula of aaHash is shown in Equation (3). $s_{i}$ denotes the *i*th *k-*mer being hashed. The base and recursive formulas are equivalent to that of ntHash (v2.3.0).


(3) $$H\left(s_{i}\right)=\mathrm{sro}l^{1}(H\left(s_{i-1}\right))\;⊕\;\mathrm{sro}l^{k}{(h}_{l}\left(s_{i-1}\left[0\right]\right))\;⊕\;h_{l}\left(s_{i}\left[k-1\right]\right)$$

To evaluate aaHash, we compared its speed, uniformity, and RAM usage against those of CityHash (commit f5dc541), MurmurHash (commit 92cf370), and xxHash (v0.8.1) (Supplementary Table S1). We also evaluated aaHash against an implementation of Rabin–Karp (commit 23b133a) to compare the hashing speed of aaHash with that of another rolling hash algorithm. All benchmarking tests were performed using a single thread on a server-class system with 144 Intel(R) Xeon(R) Gold 6254 CPU @ 3.1 GHz with 2.9 TB RAM. aaHash is freely available and implemented within the btllib common code library v1.6.0 (Nikolić *et al.* 2022). Statistical tests were conducted using python (v3.10.12) and SciPy (1.11.3) (Virtanen *et al.* 2020). aaHash documentation and the tests used to generate the results presented in this paper can be found in our GitHub repository at: https://github.com/bcgsc/aahash_paper.

## 3 Results

**Figure 1.**
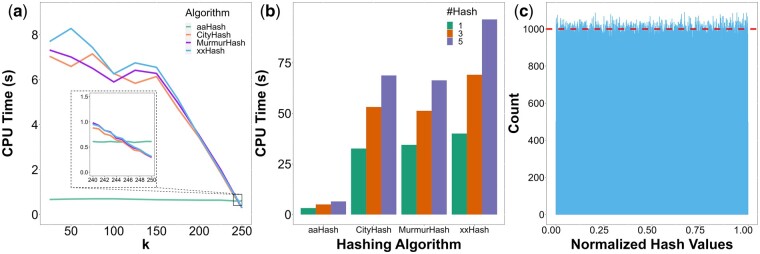
Performance of aaHash. (a) Runtime for hashing 1 000 000 × 250 amino acids residue long sequences with *k*-mer lengths from 25 to 250. aaHash outperforms all other hashing methods when computing more than five subsequent *k*-mers (i.e. *k *<* *246, see inset). (b) Comparing multi-hashing runtime of aaHash versus other state-of-the-art hashing functions for one billion 50-mers. aaHash hashing is ∼10× faster than the closest competitor, CityHash. The colours indicate the number of hashes generated. (c) Histogram of 1 000 000 100-mer hashes generated by aaHash from a random amino acid sequence of length 1 000 099. The dashed line indicates the average number of hashes in a bin (1000). The hash values were normalized by dividing the hash values by $2^{64}-1$, the largest 64-bit integer, and plotted on the histogram with bin size of 1000. The mean and standard deviation of the bin counts are 1000.0 ± 31.4, demonstrating the empirical uniformity of aaHash.

To evaluate aaHash’s performance in hashing consecutive amino acid *k*-mers, we hashed 1 000 000 random simulated peptide sequences, each with 250 amino acid residues, using aaHash and three state-of-the-art hashing algorithms, CityHash, MurmurHash, and xxHash (Supplementary Note S1). aaHash is the fastest among all competitors when hashing at least six adjacent *k*-mers, and achieves up to ∼10× speed improvement over the second fastest hashing algorithm, CityHash, (0.67 s vs 7.04 s) when hashing 226 consecutive 25-mers (Fig. 1a, Supplementary Table S2). aaHash also hashed one billion consecutive 50-mers in 3.19 s, 4.99 s, and 6.48 s to generate 1, 3, and 5 hashes per *k-*mer, respectively, again at least an order of magnitude faster than comparators in this use case (Fig. 1b, Supplementary Table S3). In a separate experiment, we compared the hashing speed of aaHash, a cyclical rolling hash algorithm against that of Rabin–Karp which employs a polynomial rolling approach (Supplementary Note S1). The results indicate that aaHash is ∼20 times faster than the Rabin–Karp algorithm across the *k* values tested (Supplementary Fig. S3, Supplementary Table S4). This large difference in hashing speed can be attributed to the computational efficiency of the rolling operations involved. Specifically, both the division and polynomial computations that Rabin–Karp utilizes are computationally more expensive than the cyclical rolls, XOR and bit-swap operations in aaHash.

Next, since uniform distribution of the hashes over the hash space leads to smaller collision probabilities, we evaluated the uniformity of the distribution of aaHash hash values by plotting a histogram of 1 000 000 normalized hash values derived from 100-mers of a random amino acid sequence (Fig. 1c). Using 1000 bins, the mean and standard deviation are 1000.0 ± 31.4, close to the ideal count of 1000 per bin. We observed similar uniformity and distribution for levels 2 and 3 hashes, achieving a mean and standard deviation of 1000.0 ± 31.2 and 1000.0 ± 32.1 (Supplementary Figs S4 and S5). A Kolmogorov–Smirnov (K-S) test was used to corroborate these observations. A significant *P-*value (with α=0.05) would indicate the rejection of the null hypothesis, suggesting that the aaHash hash distribution significantly differs from a uniform hash distribution. Our results showed that the distribution of different level hash values aaHash generated are not significantly different from a uniform distribution, an important quality of good hash functions (K-S statistics of 0.0011, 0.0010, and 0.0056 and *P*-values of 0.15, 0.27, and 0.91 for level 1, 2, and 3 hash, respectively) (Chakravarti *et al.* 1967). We also applied the Box–Muller transformation (Box and Muller 1958) to the aaHash hash values at different levels to assess their uniformity using the previous test setup (Birol *et al.* 2018). The Box–Muller transform converts two independent samples from a uniform distribution, $U_{0}$ and $U_{1}$, into two random independent variables with a standard normal distribution, $Z_{0}$ and $Z_{1}$, as shown in Equations (4) and (5).


(4) $$Z_{0}=\sqrt{-2\ln U_{0}}\cos\left(2\pi U_{1}\right)$$

(5) $$Z_{1}=\sqrt{-2\ln U_{0}\;}\sin\left(2\pi U_{1}\right)$$

Adjacent *k-*mer hashes were selected as the input to the Box–Muller transform to test for their uniformity and independence. We then plotted the quantiles of each resulting distribution against the theoretical quantiles of a normal distribution in a Q–Q plot (Wilk and Gnanadesikan 1968) (Supplementary Fig. S6). Visually, the quantiles of the transformed distributions align well with the identity line, indicating a strong similarity between the transformed and normal distributions. We then tested the normality of the transformed distributions using the Shapiro–Wilk (SW) test (Shapiro and Wilk 1965). The test reported SW test statistic of 1.00 for all distributions and *P-*values of 0.44 and 0.25, 0.11 and 0.55, 0.49 and 0.86 for $Z_{0}$ and $Z_{1}$ distributions for level 1, 2, and 3 hashes, respectively. A significant *P-*value (with α=0.05) would indicate the rejection of the null hypothesis, suggesting that the transformed distribution is significantly different from a normal distribution. A Pearson correlation coefficient (Freedman *et al.* 2007) was computed between the $Z_{0}$ and $Z_{1}$ distributions derived from the aaHash hash values at different levels. We found that correlations between the $Z_{0}$ and $Z_{1}$ distributions of the transformed level 1, 2, and 3 aaHash hash values were all consistent with zero (−0.023 ± 0.032, 0.026 ± 0.032, and −0.024 ± 0.032, respectively, where the deviation indicated is one standard error), suggesting independence between the $Z_{0}$ and $Z_{1}$ distributions. As the SW test is sensitive to large sample size, we selected the first 1000 transformed hash values, comfortably below the upper limit of 5000 recommended by SciPy, as the input for both tests. The SW test results and Pearson correlation coefficients suggest the transformed distributions are both normal and independent. As the Box–Muller transformation is a bijection, the normality and independence of the transformed values imply that the untransformed aaHash hash values are uniform and independent. Lastly, we compared the uniformity and distribution of the hash values generated by aaHash and the state-of-the-art hashing algorithms by querying *k*-mers from the UniProt human proteome (Bateman *et al.* 2023) and 1 000 000 randomly simulated 250 amino acid residues long sequences against a Bloom filter (Bloom 1970), a probabilistic data structure, loaded with *k*-mers from 1 000 000 randomly generated 250 amino acid residues long sequences (Supplementary Note S1, Supplementary Tables S5–S10). As the Bloom filter contains only randomly generated *k*-mers, any hits would be considered false positives. We then compared the actual false positive rate with the theoretical false positive rate calculated using the occupancy of the Bloom filter to determine if the aaHash-generated hash values follow a uniform distribution. aaHash achieved false positive rates of 11.9 ± 0.3%, 3.1 ± 0.07%, and 2.2 ± 0.04% for 1, 3, and 5 hashes per *k-*mer, respectively. These do not differ significantly from the theoretical false positive rates of 11.8%, 3.1%, and 2.2% for 1, 3, and 5 hashes per *k*-mer, respectively, for both datasets across multiple *k*-mer lengths based on the two-sided paired Student’s *t*-test, where a significant *P-*value (with α=0.05) would reject the null hypothesis and indicate the aaHash false positive rates significantly differ from the theoretical false positive rates (t statistics of 0.98, 1.00, and 1.00 and *P*-values of 0.37, 0.36, and 0.36) (Student 1908). These results, which simulate a possible use case of aaHash, are also on par with the other state-of-the-art hashing algorithms and demonstrate that the aaHash algorithm is empirically uniform, reaffirming the results of a previous study investigating recursive hashing (Cohen 1997).

Generic recursive cyclical *k-*mer (n*-*gram) hashing approaches can have limitations for use cases requiring larger *k-*mer sizes. In particular, they are constrained by the size of the datatype (e.g. hashing a *k*-mer longer than 64 bases is non-uniform if stored in a 64-bit integer) (Supplementary Fig. S7). The Rabin–Karp rolling hash algorithm shares a similar limitation. When *k* is sufficiently large, the value of $b^{k}$ can exceed the size of the datatype, leading to a loss in information, and thus increasing the likelihood of hash collisions. For aaHash, the bit-swap operation in Equation (2) increases the periodicity from 64 to 1023. Given that typical *k-*mer hashing applications utilize *k*-mer sizes ≪ 1023, this bit-swap mechanism renders aaHash functionally uniform for typical use cases. While a previous study established that cyclical recursive n-gram hashing is not formally uniform (Lemire and Kaser 2010), the considerations in the formulation of aaHash and the results of the empirical uniformity and independence tests demonstrate that aaHash is a suitable hashing algorithm for bioinformatics applications.

Finally, we compared the peak memory usage of each hashing algorithm and note that the peak memory usage does not differ substantially, regardless of *k*-mer length and number of hashes generated (Supplementary Tables S11 and S12).

Unlike other hashing algorithms, aaHash introduces second and third level hashing, enabling researchers to compare amino acid sequences at the hash level using the BLOSUM62 matrix. BLOSUM62 was chosen because it is the default substitution matrix for BLASTp (Altschul *et al.* 1990), but the concept can be extended to other similarity matrices like PAM matrices (Dayhoff *et al.* 1978). In addition, aaHash supports the integration of various hash levels to produce multi-level patterns. These multi-level patterns mimic the functionality of spaced seeds (a pattern with ‘care’ and ‘don’t care’ positions) (Ma *et al.* 2002), which are typically used for approximate matching in DNA homology searches, but instead of completely ignoring the ‘don’t care’ positions, aaHash will consider the biochemical similarity between the amino acids based on the hash level of each position.

aaHash is a specialized amino acid hashing algorithm that outperforms other state-of-the-art hashing algorithms in speed when hashing consecutive amino acid *k*-mers, a frequently employed operation in bioinformatics. Additionally, the implementation of multi-level hashing has great potential for enabling homology searches between evolutionarily divergent sequences. With its improved speed over other state-of-the art algorithms and homology-oriented features, we expect aaHash to be both beneficial to the scientific community and improve many bioinformatics applications involving amino acid sequence analysis.

## Supplementary Material

vbad162_Supplementary_Data

## Data Availability

Information about the data used to benchmark aaHash and its comparators can be found in Supplementary Note S1.
