# Supplementary material for: aaHash: recursive amino acid sequence hashing
Source: Bioinform Adv. 2023 Nov 11;3(1):vbad162. doi: 10.1093/bioadv/vbad162 (PMC10660294; doi:10.1093/bioadv/vbad162)
Supplement: vbad162_Supplementary_Data [file vbad162_supplementary_data.zip › aaHash_supplementary.pdf]

Supplementary Information for: aaHash: recursive amino acid sequence hashing

Johnathan Wong, Parham Kazemi, Lauren Coombe, René L. Warren, Inanç Birol

Table of Contents

Supplementary Note S1: aaHash benchmarking data 3

Supplementary Figure S1. Using pre-built seed tables to compute the base hash value........ 4

Supplementary Figure S2. Sorted BLOSUM62 matrix........ 5

Supplementary Figure S3. Runtime for hashing 1,000,000 x 250 amino acids residue long sequences with *k*-mer lengths from 25 to 250 using aaHash or Rabin-Karp. 6

Supplementary Figure S4. Histogram of 1,000,000 100-mer level 2 hashes generated by aaHash from a random amino acid sequence of length 1,000,099........ 7

Supplementary Figure S5. Histogram of 1,000,000 100-mer level 3 hashes generated by aaHash from a random amino acid sequence of length 1,000,099........ 8

Supplementary Figure S6. Q-Q plots generated from 500,000 Box-Muller transformed levels 1, 2, and 3 aaHash 100-mer hash values. 9

Supplementary Figure S7. Periodicity demonstration of cyclical hashing........ 10

Supplementary Table S1. Hashing algorithms used to benchmark aaHash. 11

Supplementary Table S3. Run time evaluation for aaHash and comparators on simulated data generating 1, 3, or 5 hash values for 1,000,000,000 adjacent amino acid 50-mers........ 11

Supplementary Table S5. Bloom filter evaluation for aaHash and comparators on experimental data using a single hash. 12

Supplementary Table S6. Bloom filter evaluation for aaHash and comparators on experimental data using 3 hashes. 12

Supplementary Table S7. Bloom filter evaluation for aaHash and comparators on experimental data using 5 hashes. 13

Supplementary Table S8. Bloom filter evaluation for aaHash and comparators on simulated data using a single hash. 13

Supplementary Table S9. Bloom filter evaluation for aaHash and comparators on simulated data using 3 hashes. 14

Supplementary Table S10. Bloom filter evaluation for aaHash and comparators on simulated data using 5 hashes. 14

Supplementary Table S11. Benchmarking script peak memory usage of each hashing algorithm generating 1 hash value for each *k*-mer with different *k*-mer lengths from 1,000,000 simulated sequences of length 250 amino acid residues........ 15

Supplementary Table S12. Benchmarking script peak memory usage of each hashing algorithm generating 1, 3, or 5 hash values for each *k*-mer with *k*=50 from 1,000,000 simulated sequences of length 250 amino acid residues. 15

Supplementary References 16

Supplementary Note S1: aaHash benchmarking data

The dataset we used to generate Supplementary Tables S5-7 are from the UniProt human canonical and isoform proteome (Bateman et al., 2023). The file can be downloaded using this hyperlink: https://doi.org/10.5281/zenodo.7793251.

For Supplementary Figs. S3-6, and Supplementary Tables S2-4, S8-12, we used random amino acid sequences generated using the btllib library (Nikolić et al., 2022).

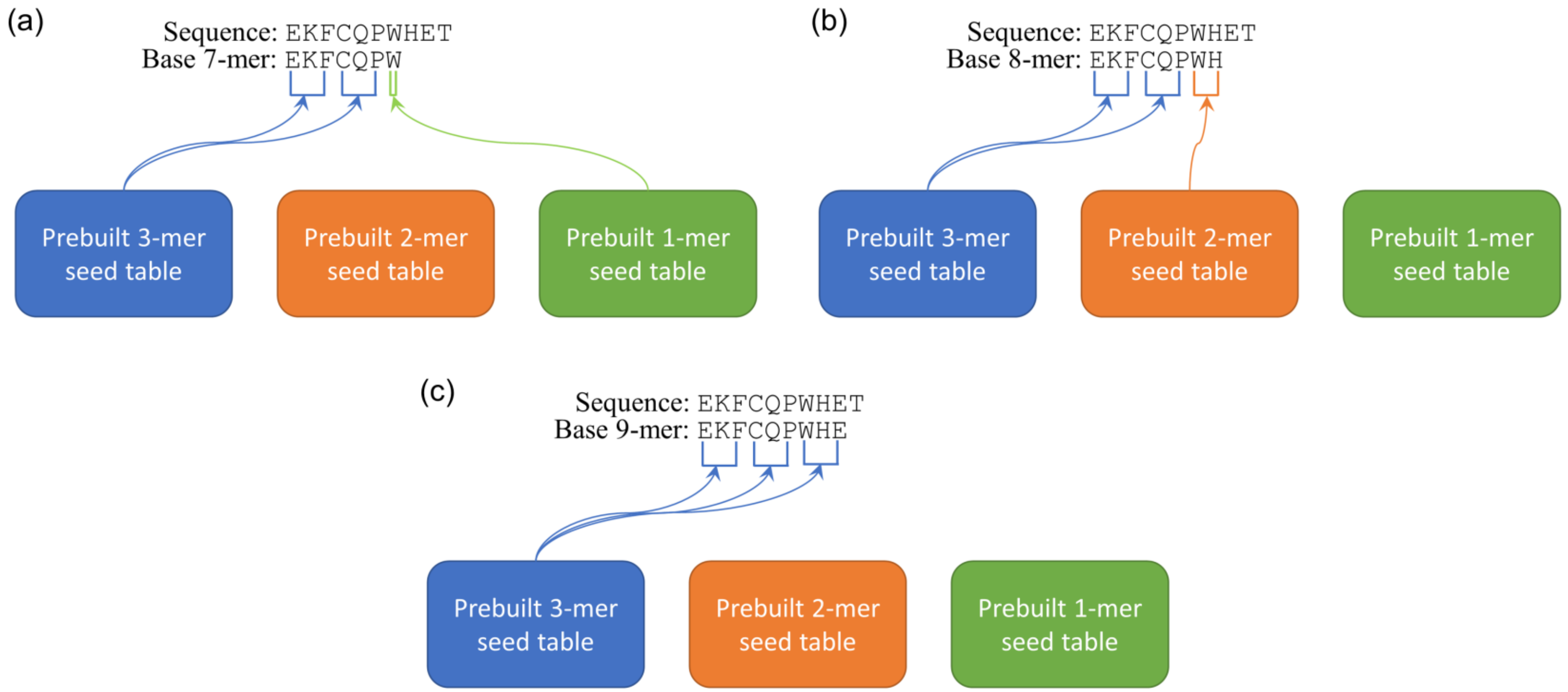

Supplementary Figure S1. Using pre-built seed tables to compute the base hash value. (a) Generating base 7-mer hash. **(b)** Generating base 8-mer hash. **(c)** Generating base 9-mer hash. By using prebuilt seed tables, aaHash reduces the number of operations required to create a base hash from k, where k is the size of the k-mer, to $\lceil k \div 3 \rceil$.

	C	G	A	T	S	N	D	E	Q	K	R	V	I	L	M	W	F	Y	H	P
**C**	9	-3	0	-1	-1	-3	-3	-4	-3	-3	-3	-1	-1	-1	-1	-2	-2	-2	-3	-3
**G**	-3	6	0	-2	0	0	-1	-2	-2	-2	-2	-3	-4	-4	-3	-2	-3	-3	-2	-2
**A**	0	0	4	0	1	-2	-2	-1	-1	-1	-1	0	-1	-1	-1	-3	-2	-2	-2	-1
**T**	-1	-2	0	5	1	0	-1	-1	-1	-1	-1	0	-1	-1	-1	-2	-2	-2	-2	-1
**S**	-1	0	1	1	4	1	0	0	0	0	-1	-2	-2	-2	-1	-3	-2	-2	-1	-1
**N**	-3	0	-2	0	1	6	1	0	0	0	0	-3	-3	-3	-2	-4	-3	-2	1	-2
**D**	-3	-1	-2	-1	0	1	6	2	0	-1	-2	-3	-3	-4	-3	-4	-3	-3	-1	-1
**E**	-4	-2	-1	-1	0	0	2	5	2	1	0	-2	-3	-3	-2	-3	-3	-2	0	-1
**Q**	-3	-2	-1	-1	0	0	0	2	5	1	1	-2	-3	-2	0	-2	-3	-1	0	-1
**K**	-3	-2	-1	-1	0	0	-1	1	1	5	2	-2	-3	-2	-1	-3	-3	-2	-1	1
**R**	-3	-2	-1	-1	-1	0	-2	0	1	2	5	-3	-3	-2	-1	-3	-3	-2	0	-2
**V**	-1	-3	0	0	-2	-3	-3	-2	-2	-2	-3	4	3	1	1	-3	-1	-1	-3	-2
**I**	-1	-4	-1	-1	-2	-3	-3	-3	-3	-3	-3	3	4	2	1	-3	0	-1	-3	-3
**L**	-1	-4	-1	-1	-2	-3	-4	-3	-2	-2	-2	1	2	4	2	-2	0	-1	-3	-3
**M**	-1	-3	-1	-1	-1	-2	-3	-2	0	-1	-1	1	1	2	5	-1	0	-1	-2	-2
**W**	-2	-2	-3	-2	-3	-4	-4	-3	-2	-3	-3	-3	-3	-2	-1	11	1	2	-2	-4
**F**	-2	-3	-2	-2	-2	-3	-3	-3	-3	-3	-3	-1	0	0	0	1	6	3	-1	-4
**Y**	-2	-3	-2	-2	-2	-2	-3	-2	-1	-2	-2	-1	-1	-1	-1	2	3	7	2	-3
**H**	-3	-2	-2	-2	-1	1	-1	0	0	-1	0	-3	-3	-3	-2	-2	1	2	8	-2
**P**	-3	-2	-1	-1	-1	-2	-1	-1	-1	-1	-2	-2	-3	-3	-2	-4	-4	-3	-2	7

Supplementary Figure S2. Sorted BLOSUM62 matrix. The BLOSUM (Henikoff & Henikoff, 1992) matrix is sorted such that positive scores, denoting similarity between amino acids, are located on the diagonal. This allows us to cluster similar amino acids together. These clusters are then used to determine which amino acids share the same hash value. The yellow highlighted squares represent which amino acids are hashed to the same value in the level 2 hash (Li et al., 2009). The blue outlined squares represent which amino acids are hashed to the same value in the level 3 hash.

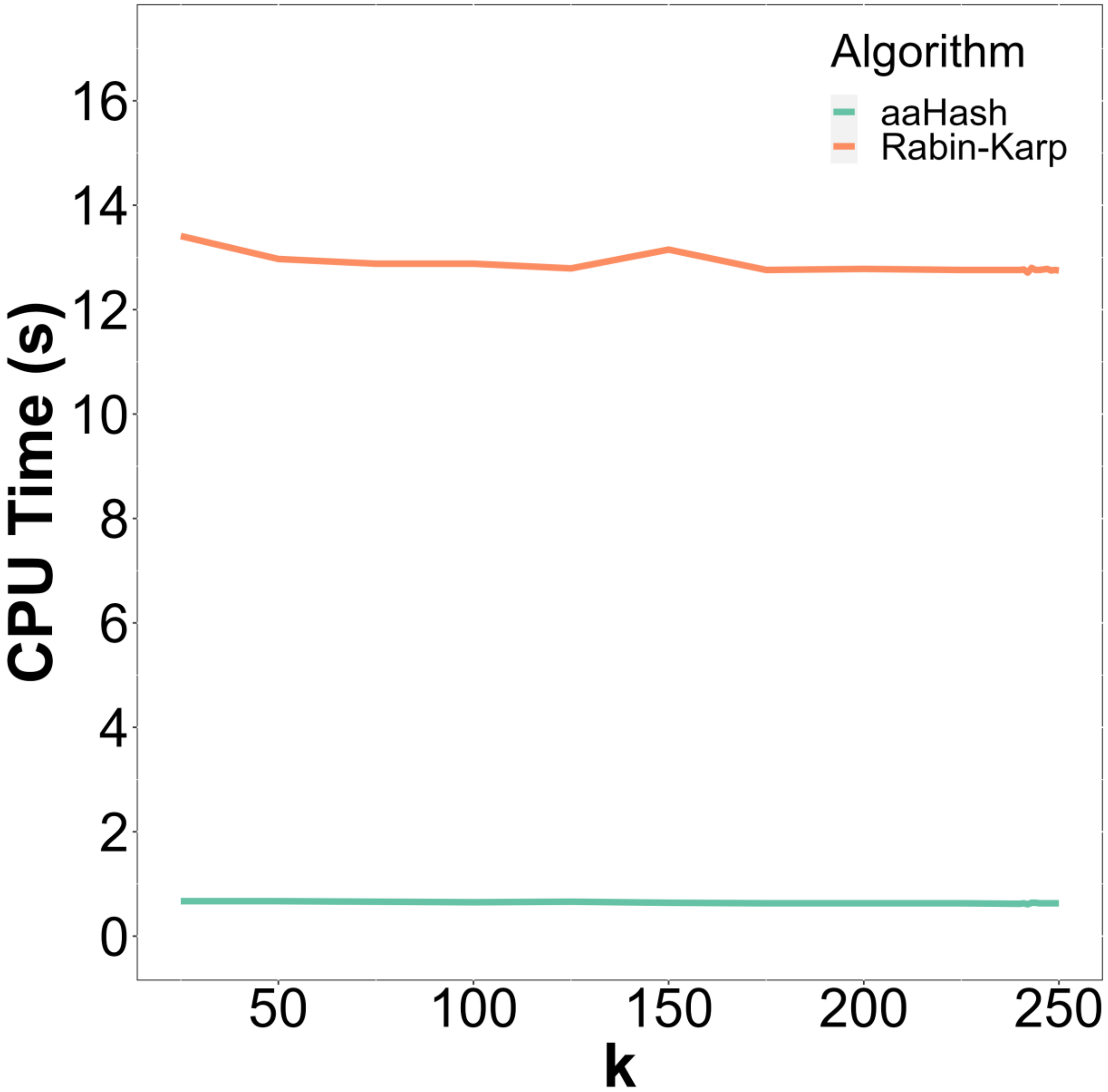

Supplementary Figure S3. Runtime for hashing 1,000,000 x 250 amino acids residue long sequences with *k*-mer lengths from 25 to 250 using aaHash or Rabin-Karp. For all tested k values, aaHash outperforms Rabin-Karp by a factor of 20.

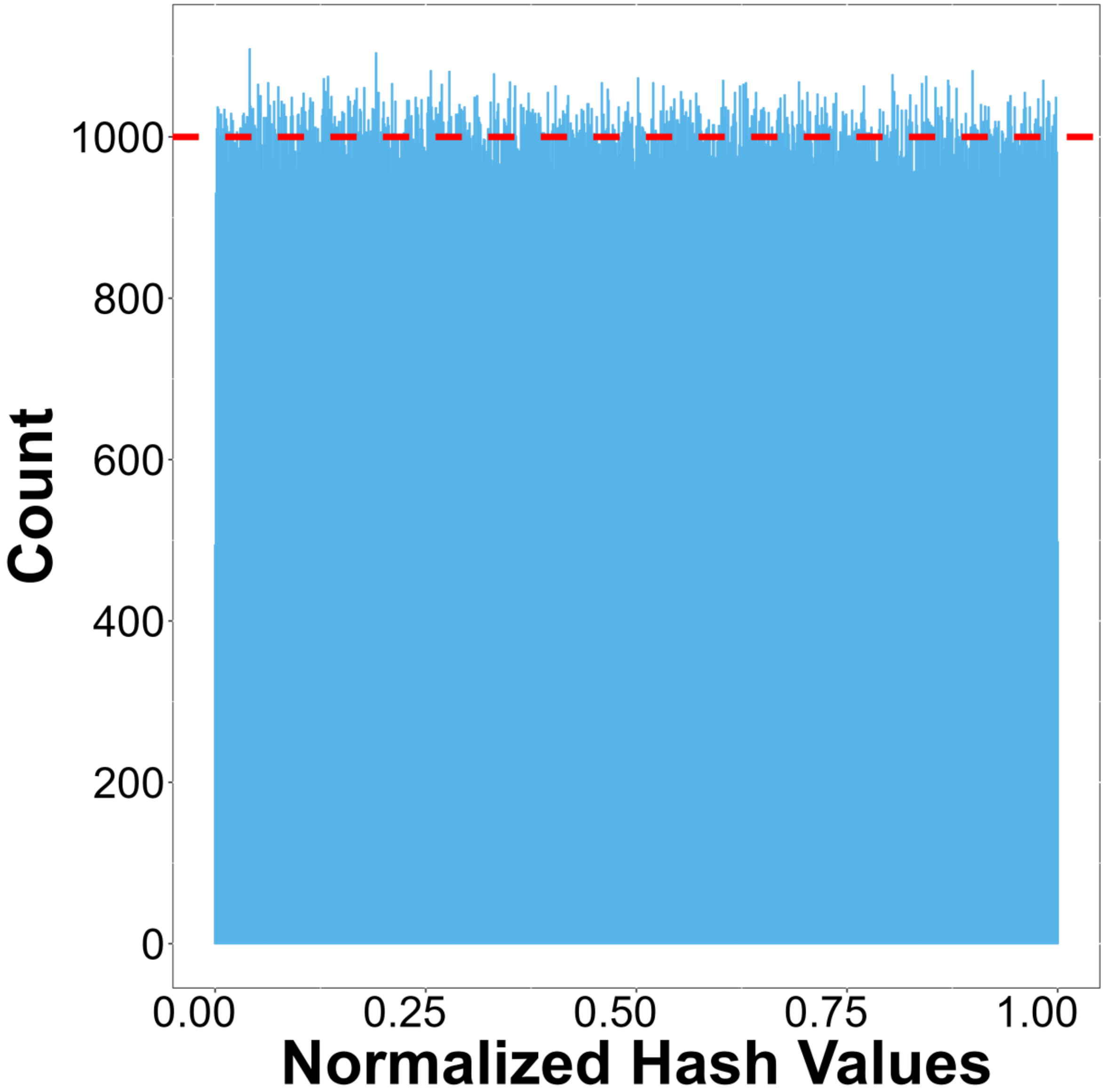

Supplementary Figure S4. Histogram of 1,000,000 100-mer level 2 hashes generated by aaHash from a random amino acid sequence of length 1,000,099. The red dashed line indicates the average number of hashes in a bin (1,000). The hash values were normalized by dividing them with $2^{64} - 1$, the largest 64-bit integer, and plotted on the histogram with bin size of 1,000. The mean and standard deviation of the bin counts are 1000.0±31.2, demonstrating the uniformity of aaHash.

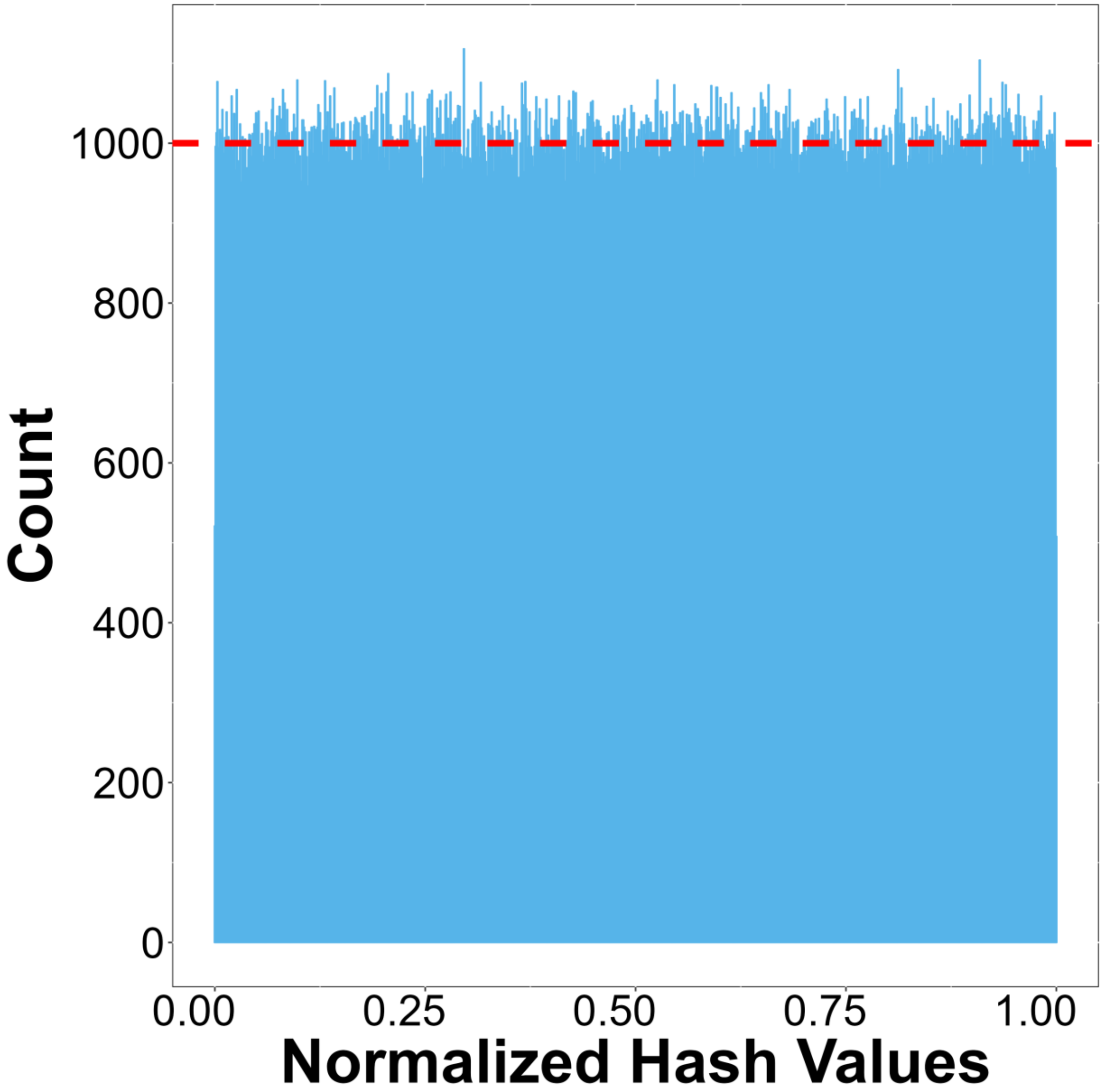

Supplementary Figure S5. Histogram of 1,000,000 100-mer level 3 hashes generated by aaHash from a random amino acid sequence of length 1,000,099. The red dashed line indicates the average number of hashes in a bin (1,000). The hash values were normalized by dividing them with $2^{64} - 1$, the largest 64-bit integer, and plotted on the histogram with bin size of 1,000. The mean and standard deviation of the bin counts are 1000.0±32.1, demonstrating the uniformity of aaHash.

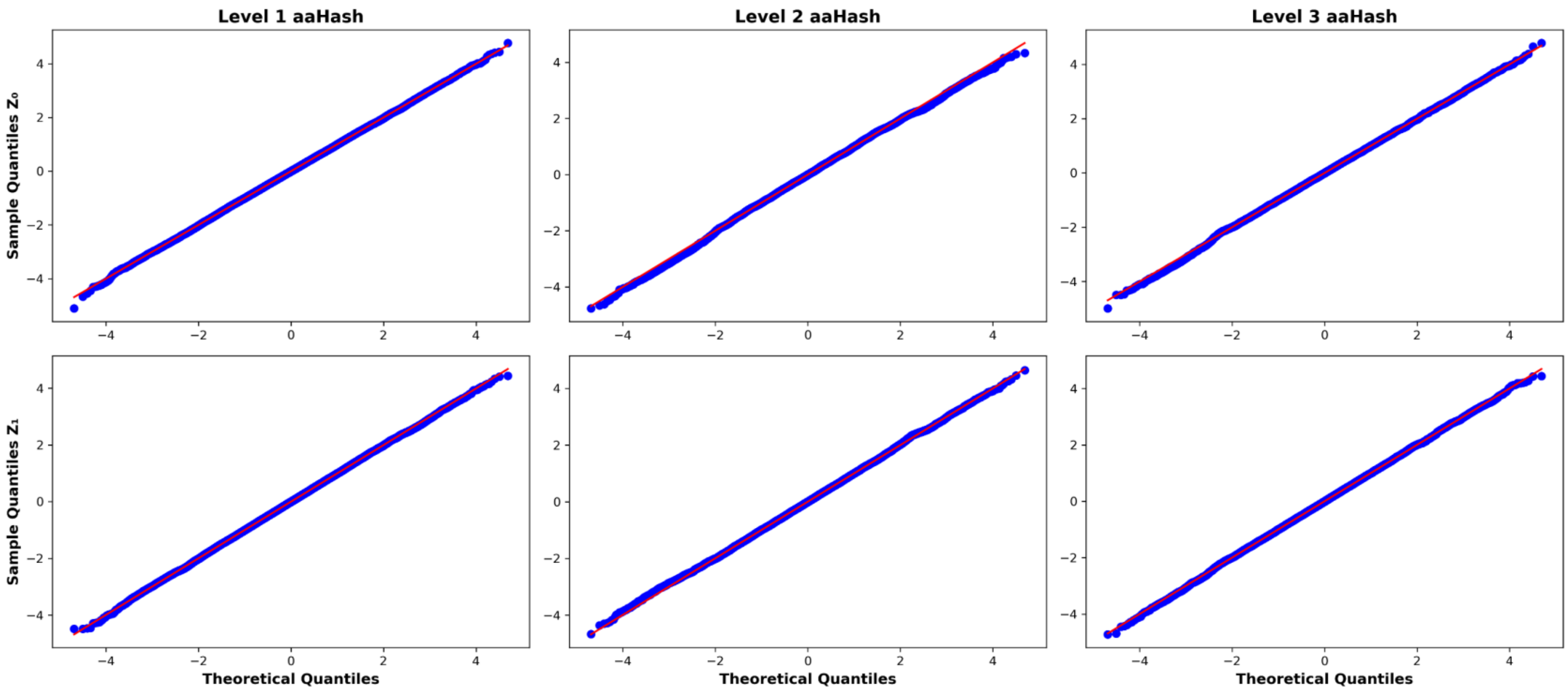

Supplementary Figure S6. Q-Q plots generated from 500,000 Box-Muller transformed levels 1, 2, and 3 aaHash 100-mer hash values. Deviations from the identity line indicate discrepancies from the theoretical normal distribution. The sample quantiles closely align with the theoretical quantiles, indicating normality.

5-mer	abcde	ebcda
5-mer cyclical hash construction	0x000a<br>0x00ab<br>0x0abc<br>0xabcd<br>0xbcde XOR 0x000a	0x000e<br>0x00eb<br>0x0ebc<br>0xebcd<br>0xbcda XOR 0x000e
5-mer hash value	48340	48340

Supplementary Figure S7. Periodicity demonstration of cyclical hashing. The datatype used for this hash is a 16-bit integer. Each character is hashed to its equivalent hexadecimal value. The hash values are also represented in hexadecimal format. With each cyclical roll shifting by 4 bits, the end result produces equivalent hash values for the two different 5-mers.

Supplementary Table S1. Hashing algorithms used to benchmark aaHash. All benchmarking tests were performed using 1 thread on a server-class system with 144 Intel(R) Xeon(R) Gold 6254 CPU @ 3.1 GHz with 2.9 TB RAM. Commit of files are listed when there is no release version of the repository.

Algorithm	Code repository	Version/commit
City hash	https://github.com/google/cityhash	f5dc541
Murmur hash	https://github.com/aappleby/smhasher	92cf370
xxHash	https://github.com/Cyan4973/xxHash	0.8.1
Rabin-Karp	https://github.com/TheAlgorithms/C-Plus-Plus	23b133a

Supplementary Table S3. Run time evaluation for aaHash and comparators on simulated data generating 1, 3, or 5 hash values for 1,000,000,000 adjacent amino acid 50-mers.

Algorithm	Number of hashes	Time (s)
aaHash	1	3.19
CityHash		32.63
MurmurHash		34.45
xxHash		40.04
aaHash	3	4.99
CityHash		53.17
MurmurHash		51.27
xxHash		69.10
aaHash	5	6.48
CityHash		68.77
MurmurHash		66.35
xxHash		96.47

Supplementary Table S5. Bloom filter evaluation for aaHash and comparators on experimental data using a single hash. We allocate 8 bits of memory per *k*-mer based on the expected number of *k*-mers in the insertion file and load the Bloom filter with a file containing 1,000,000 randomly generated sequences of length 250 amino acid residues. Then, we query the Bloom filter with the human proteome containing all canonical forms and isoforms with *k*-mers of sizes 50, 150 and 250. The theoretical approximate false positive rate is 11.8%.

Algorithm	*k*-mer size	Number of Queries	Set bits in BF	False hits	False Positive Rate (%)
aaHash	50	22,240,331	188,943,234	2,614,647	11.7
CityHash			188,945,163	2,612,903	11.8
MurmurHash			188,945,552	2,614,662	11.8
xxHash			18,8947,318	2,615,142	11.8
aaHash	150	18,005,231	94,943,041	2,135,966	11.9
CityHash			94,940,478	2,138,626	11.9
MurmurHash			94,938,194	2,134,078	11.9
xxHash			94,947,078	2,138,659	11.9
aaHash	250	13,770,131	939,841	1,725,160	12.5
CityHash			939,981	1,727,276	12.5
MurmurHash			939,866	1,724,809	12.5
xxHash			940,057	1,7281,30	12.6

Supplementary Table S6. Bloom filter evaluation for aaHash and comparators on experimental data using 3 hashes. We allocate 8 bits of memory per *k*-mer based on the expected number of *k*-mers in the insertion file and load the Bloom filter with a file containing 1,000,000 randomly generated sequences of length 250 amino acid residues. Then, we query the Bloom filter with the human proteome containing all canonical forms and isoforms with *k*-mers of sizes 50, 150 and 250. The theoretical approximate false positive rate is 3.1%.

Algorithm	*k*-mer size	Number of Queries	Set bits in BF	False hits	False Positive Rate (%)
aaHash	50	22,240,331	502,823,792	680,002	3.1
CityHash			502,848,163	680,632	3.1
MurmurHash			502,841,165	676,344	3.0
xxHash			502,840,832	680,413	3.1
aaHash	150	18,005,231	252,675,404	555,191	3.1
CityHash			252,672,915	556,088	3.1
MurmurHash			252,675,830	553,780	3.1
xxHash			252,679,859	557,243	3.1
aaHash	250	13,770,131	2,502,111	448,559	3.3
CityHash			2,501,524	450,201	3.3
MurmurHash			2,501,649	447,992	3.3
xxHash			2,502,508	450,084	3.3

Supplementary Table S7. Bloom filter evaluation for aaHash and comparators on experimental data using 5 hashes. We allocate 8 bits of memory per *k*-mer based on the expected number of *k*-mers in the insertion file and load the Bloom filter with a file containing 1,000,000 randomly generated sequences of length 250 amino acid residues. Then, we query the Bloom filter with the human proteome containing all canonical forms and isoforms with *k*-mers of sizes 50, 150 and 250. The theoretical approximate false positive rate is 2.2%.

Algorithm	*k*-mer size	Number of Queries	Set bits in BF	False hits	False Positive Rate (%)
aaHash	50	22,240,331	747,287,691	482,467	2.2
CityHash			747,321,437	482,516	2.2
MurmurHash			747,312,591	482,472	2.2
xxHash			747,299,027	480,699	2.2
aaHash	150	18,005,231	375,509,789	394,163	2.2
CityHash			375,509,620	395,910	2.2
MurmurHash			375,508,139	392,860	2.2
xxHash			375,509,658	393,850	2.2
aaHash	250	13,770,131	3,717,896	318,749	2.3
CityHash			3,718,154	318,951	2.3
MurmurHash			3,717,539	317,164	2.3
xxHash			3,719,204	318,731	2.3

Supplementary Table S8. Bloom filter evaluation for aaHash and comparators on simulated data using a single hash. We allocate 8 bits of memory per *k*-mer based on the expected number of *k*-mers in the insertion file and load the Bloom filter with a file containing 1,000,000 randomly generated sequences of length 250 amino acid residues. Then, we query the Bloom filter with the 1,000,000 simulated sequences of length 250 amino acid residues with *k*-mers of sizes 50, 150 and 250. The theoretical approximate false positive rate is 11.8%.

Algorithm	*k*-mer size	Number of Queries	Set bits in BF	False hits	False Positive Rate (%)
aaHash	50	201,000,000	188,943,234	23,616,753	11.8
CityHash			188,945,163	23,619,243	11.8
MurmurHash			188,945,552	23,619,240	11.8
xxHash			18,8947,318	23,624,417	11.8
aaHash	150	101,000,000	94,943,041	11,867,803	11.8
CityHash			94,940,478	11,865,281	11.8
MurmurHash			94,938,194	11,866,971	11.8
xxHash			94,947,078	11,869,525	11.8
aaHash	250	1,000,000	939,841	117,478	11.8
CityHash			939,981	117,903	11.8
MurmurHash			939,866	117,180	11.7
xxHash			940,057	117,503	11.8

Supplementary Table S9. Bloom filter evaluation for aaHash and comparators on simulated data using 3 hashes. We allocate 8 bits of memory per *k*-mer based on the expected number of *k*-mers in the insertion file and load the Bloom filter with a file containing 1,000,000 randomly generated sequences of length 250 amino acid residues. Then, we query the Bloom filter with the 1,000,000 simulated sequences of length 250 amino acid residues with *k*-mers of sizes 50, 150 and 250. The theoretical approximate false positive rate is 3.1%.

Algorithm	*k*-mer size	Number of Queries	Set bits in BF	False hits	False Positive Rate (%)
aaHash	50	201,000,000	502,823,792	6,146,485	3.1
CityHash			502,848,163	6,148,676	3.1
MurmurHash			502,841,165	6,144,972	3.1
xxHash			502,840,832	6,146,218	3.1
aaHash	150	101,000,000	252,675,404	3,088,838	3.1
CityHash			252,672,915	3,086,423	3.1
MurmurHash			252,675,830	3,088,088	3.1
xxHash			252,679,859	3,088,804	3.1
aaHash	250	1,000,000	2,502,111	30,466	3.1
CityHash			2,501,524	30,812	3.1
MurmurHash			2,501,649	30,450	3.1
xxHash			2,502,508	30,483	3.1

Supplementary Table S10. Bloom filter evaluation for aaHash and comparators on simulated data using 5 hashes. We allocate 8 bits of memory per *k*-mer based on the expected number of *k*-mers in the insertion file and load the Bloom filter with a file containing 1,000,000 randomly generated sequences of length 250 amino acid residues. Then, we query the Bloom filter with the 1,000,000 simulated sequences of length 250 amino acid residues with *k*-mers of sizes 50, 150 and 250. The theoretical approximate false positive rate is 2.2%.

Algorithm	*k*-mer size	Number of Queries	Set bits in BF	False hits	False Positive Rate (%)
aaHash	50	201,000,000	747,287,691	4,355,434	2.2
CityHash			747,321,437	4,359,290	2.2
MurmurHash			747,312,591	4,356,975	2.2
xxHash			747,299,027	4,355,402	2.2
aaHash	150	101,000,000	375,509,789	2,188,433	2.2
CityHash			375,509,620	2,187,385	2.2
MurmurHash			375,508,139	2,189,370	2.2
xxHash			375,509,658	2,188,529	2.2
aaHash	250	1,000,000	3,717,896	21,807	2.2
CityHash			3,718,154	21,536	2.2
MurmurHash			3,717,539	21,639	2.2
xxHash			3,719,204	21,401	2.1

Supplementary Table S11. Benchmarking script peak memory usage of each hashing algorithm generating 1 hash value for each *k*-mer with different *k*-mer lengths from 1,000,000 simulated sequences of length 250 amino acid residues.

Algorithm	*k*-mer size	Peak memory usage (MB)
aaHash	50	2.1
CityHash		2.1
MurmurHash		2.1
xxHash		2.1
aaHash	150	2.1
CityHash		2.1
MurmurHash		2.1
xxHash		2.1
aaHash	250	2.1
CityHash		2.1
MurmurHash		2.1
xxHash		2.1

Supplementary Table S12. Benchmarking script peak memory usage of each hashing algorithm generating 1, 3, or 5 hash values for each *k*-mer with *k*=50 from 1,000,000 simulated sequences of length 250 amino acid residues.

Algorithm	Number of Hashes	Peak memory usage (MB)
aaHash	1	2.1
CityHash		2.1
MurmurHash		2.1
xxHash		2.1
aaHash	3	2.1
CityHash		2.1
MurmurHash		2.1
xxHash		2.1
aaHash	5	2.1
CityHash		2.1
MurmurHash		2.1
xxHash		2.1

Supplementary References

Bateman, A., Martin, M.-J., Orchard, S., Magrane, M., Ahmad, S., Alpi, E., Bowler-Barnett, E. H., Britto, R., Bye-A-Jee, H., Cukura, A., Denny, P., Dogan, T., Ebenezer, T., Fan, J., Garmiri, P., da Costa Gonzales, L. J., Hatton-Ellis, E., Hussein, A., Ignatchenko, A., … Zhang, J. (2023). UniProt: the Universal Protein Knowledgebase in 2023. *Nucleic Acids Research*, *51*(D1), D523–D531. https://doi.org/10.1093/nar/gkac1052

Henikoff, S., & Henikoff, J. G. (1992). Amino acid substitution matrices from protein blocks. *Proceedings of the National Academy of Sciences*, *89*(22), 10915–10919. https://doi.org/10.1073/pnas.89.22.10915

Li, W., Ma, B., & Zhang, K. (2009). Amino Acid Classification and Hash Seeds for Homology Search. In S. Rajasekaran (Ed.), *Bioinformatics and Computational Biology* (pp. 44–51). Springer Berlin Heidelberg.

Nikolić, V., Kazemi, P., Coombe, L., Wong, J., Afshinfard, A., Chu, J., Warren, R. L., & Birol, I. (2022). btllib: A C++ library with Python interface for efficient genomic sequence processing. *Journal of Open Source Software*, *7*(79), 4720. https://doi.org/10.21105/joss.04720
